# Extensive amyloid deposits in bone marrow aspirate smears

**DOI:** 10.1002/ccr3.3236

**Published:** 2020-08-30

**Authors:** Ting Hon Stanford Li, Kit Fai Wong, Wai Shan Wong

**Affiliations:** ^1^ Department of Pathology Queen Elizabeth Hospital Hong Kong China

**Keywords:** amyloidosis

## Abstract

Amyloid light‐chain (AL) amyloidosis is the most common form of systemic amyloidosis. It can cause progressive organ dysfunction and eventually death, mainly due to cardiac involvement. Amyloidosis may rarely present as extensive amorphous, purplish‐blue deposits in marrow aspirate smears. Demonstration of congophilic property and apple‐green birefringence under polarized light in aspirate smears can allow a rapid diagnosis of amyloidosis.

## Graphical abstract

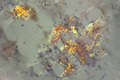

## 1 INTRODUCTION

**Figure 1:**
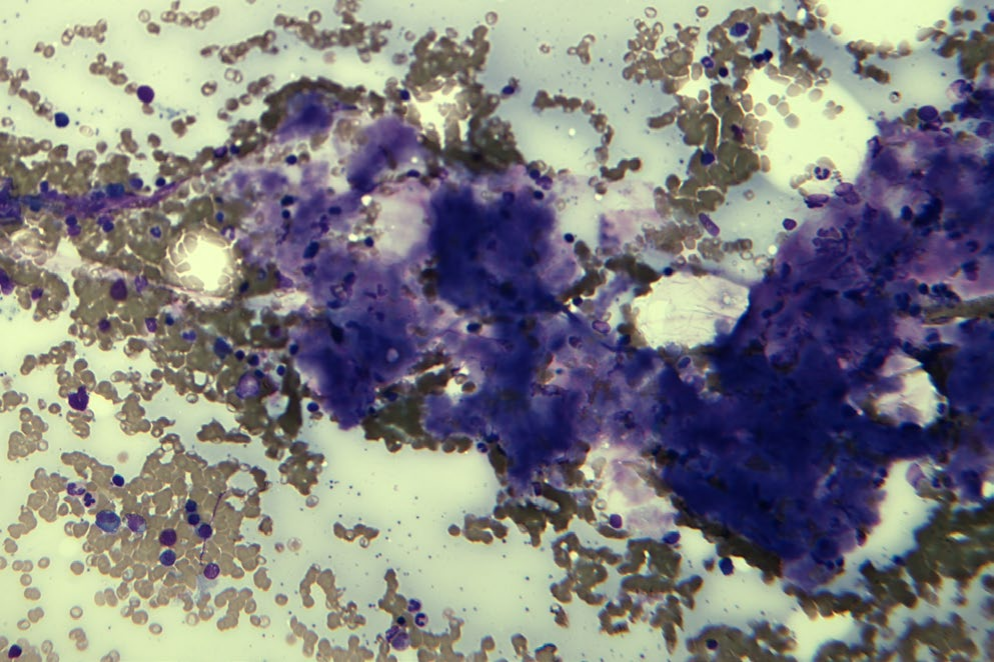
Extensive amyloid deposition in marrow aspirate smear which appeared as amorphous, purplish‐blue material

**Figure 2:**
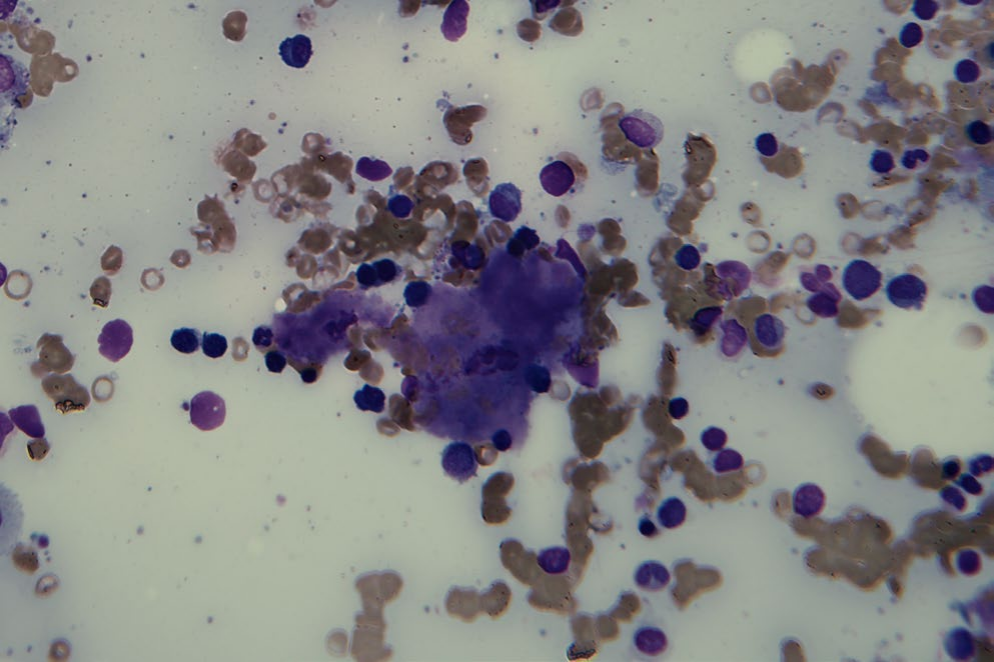
Extensive amyloid deposition in marrow aspirate smear which appeared as amorphous, purplish‐blue material

**Figure 3:**
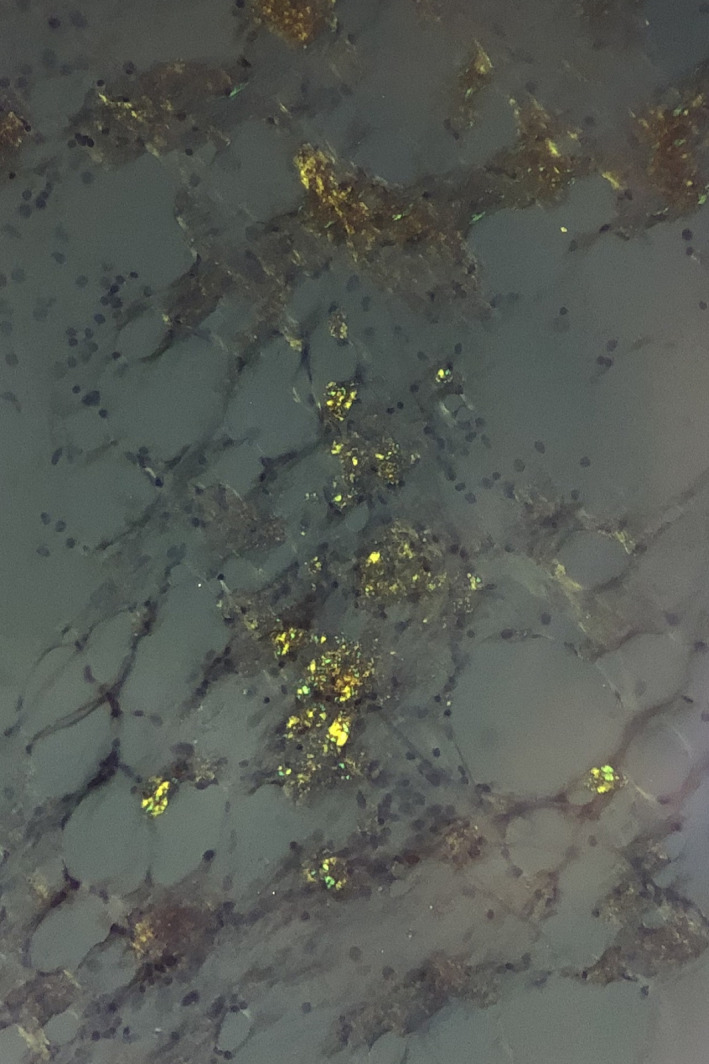
Amyloid deposits in marrow aspirate smear which were congophilic and showed apple‐green birefringence under polarized light

**Figure 4:**
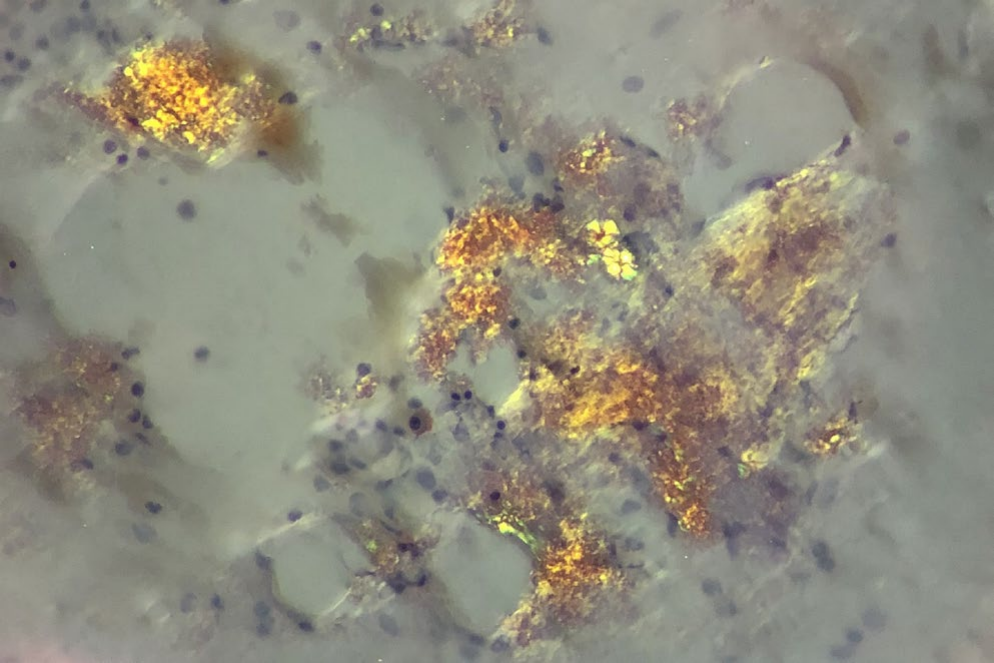
Amyloid deposits in marrow aspirate smear which were congophilic and showed apple‐green birefringence under polarized light

A 60‐year‐old Chinese man, with history of acute monoblastic leukemia in remission for 8 years, presented with bilateral lower limb swelling and frothy urine. The creatinine level was 117 µmol/L, and 24‐hr urine protein test showed proteinuria of 1.22 g/d. Serum protein electrophoresis did not demonstrate monoclonal M‐band but serum immunoglobulin‐free light chain assay showed a skewed kappa to lambda ratio of 0.12 (normal 0.26‐1.65). Renal biopsy revealed Amyloid light‐chain (AL) amyloidosis. Bone marrow examination was performed. Aspirate smears showed plasmacytosis (20%), with extensive deposition of amorphous, purplish‐blue material (Figures 1&2). The deposits in aspirate smears were congophilic and showed apple‐green birefringence under polarized light (Figures 3&4), which is pathognomonic for amyloidosis. Trephine biopsy showed plasmacytosis with a mild preponderance of lambda‐positive plasma cells over kappa‐positive plasma cells (kappa to lambda ratio = 1:3). Amyloid deposits were detected in the thickened vasculatures, which were confirmed by Congo red staining. The patient was treated with one cycle of bortezomib‐dexamethasone. He was found arrest a few days after discharge and eventually died.

AL amyloidosis is the most common form of systemic amyloidosis. It is caused by extracellular deposits of monoclonal immunoglobulin light chain produced by neoplastic plasma cells. AL amyloidosis can cause progressive organ dysfunction and eventually death, mainly due to cardiac involvement.

The presence of amyloid deposits in bone marrow is usually detected in trephine biopsy sections, most commonly in vessel walls and sometimes in the interstitium.Amyloid is usually not present or is very sparse in aspirate in majority of the cases.^1^, ^2^ Demonstration of extensive amyloid deposits with congophilic property and apple‐green birefringence under polarized light in marrow aspirate smears, as in our case, is rarely reported to our best knowledge. This finding is not only interesting in its own right, but can also allow a quick diagnosis of amyloidosis if trephine biopsy sections are not available.

## CONFLICT OF INTEREST

None declared.

## AUTHOR CONTRIBUTIONS

THSL: analyzed and interpreted the patient's data, performed the morphological examination of the peripheral blood film/ marrow aspirate, produced the images used in the manuscript, and wrote the manuscript. KFW: analyzed and interpreted the patient's data and performed the morphological examination of the peripheral blood film/ marrow aspirate. WSW: analyzed and interpreted the patient's data and performed the morphological examination of the trephine biopsy.
